# Performance Parameters in Competitive Alpine Skiing Disciplines of Slalom, Giant Slalom and Super-Giant Slalom

**DOI:** 10.3390/ijerph18052628

**Published:** 2021-03-05

**Authors:** Lidia B. Alejo, Jaime Gil-Cabrera, Almudena Montalvo-Pérez, David Barranco-Gil, Jaime Hortal-Fondón, Archit Navandar

**Affiliations:** 1 Faculty of Sports Sciences, Universidad Europea de Madrid, C/Tajo, s/n, 28670 Madrid, Spain; lidia.brea@universidadeuropea.es (L.B.A.); jaime.gil@universidadeuropea.es (J.G.-C.); almudena.montalvo@universidadeuropea.es (A.M.-P.); david.barranco@universidadeuropea.es (D.B.-G.); jaimehortal97@gmail.com (J.H.-F.); 2 Instituto de Investigación Hospital 12 de Octubre (imas12), 28041 Madrid, Spain; 3 Royal Spanish Winter Sports Federation, 28703 San Sebastian de los Reyes, Spain

**Keywords:** winter sports, impacts, GPS technology, GNSS systems, competition testing

## Abstract

The objective of this study was to describe the kinematic patterns and impacts in male and female skiers in the super-giant slalom, giant slalom and slalom disciplines of an international alpine skiing competition using a portable Global Navigation Satellite Systems (GNSS) technology device. Fifteen skiers (males, *n* = 9, females, *n* = 6) volunteered to participate in this study. Data acquisition was carried out using a wireless inertial measurement device (WIMUTM PRO: hybrid location system GNSS at 18 Hz with a precision locator UltraWideband UWD (<10 cm) and 3D accelerometers 1000 Hz) where distances covered in different speed and acceleration thresholds and impacts above 5g were recorded in each of the disciplines. Male and female alpine skiers showed different physical parameters and impacts even though they competed in the same courses in the disciplines of slalom, giant slalom and super-giant slalom (total impacts: *p* < 0.001; impacts > 7 g: *p* = 0.013; impacts 6.1–7 g: *p* = 0.002; impacts 5.1–6 g: *p* = 0.006). In male skiers, the distances traveled at different speed thresholds have a direct relation to the ranking of skiers, but this ideal threshold decreased as the technicality of the discipline increased. In the case of female skiers, although no relation was seen with the speed thresholds, greater distances covered at medium accelerations improved skiing performance. The external load in alpine skiing varied based on sex and discipline. This information could be essential to develop sex-specific and discipline-specific training programs in alpine skiing.

## 1. Introduction

Winter sports, and specifically alpine skiing, have increased in popularity all over the world, with around 400 million people worldwide visiting a ski resort in 2019 [1]. Skiing has formed an important part of Winter Olympics, with different events such as alpine skiing, cross-country skiing, ski jumping, nordic combined, freestyle skiing and snowboarding forming a part of the Olympic discipline [2]. Among them, alpine skiing, is one of the oldest forms of skiing, being a part of the Winter Olympics since 1924 and is one of the most popular of all the skiing disciplines [3].

In alpine skiing competitions, participants ski downhill in a course approved by the International Skiing Federation (FIS, Fédération Internationale de Ski), and their objective is to cover the course in the shortest time possible, with winning margins being very small [3]. The course consists of various turns determined by the position of poles or gates set at certain distances and the participants cannot skip any of the gates. The alpine skiing competition consists of three main events: slalom, giant slalom downhill, and the super giant slalom, for both female and male participants (Table 1). The slalom is the alpine event with the shortest course and the most turns. The giant slalom has fewer turns and wider, smoother turns compared to the slalom, while the super giant slalom and downhill are the longest events with the greatest vertical drops requiring both speed and precision in the technical turns [2]. Hence, it is evident that these four events have different demands at the technical and physical level [4]. An in-depth knowledge of these demands could help both skiers and coaches improve their performance by adapting training exactly to the demands of each event in competition. Although various studies have studied the physiological and biomechanical demands, a majority of them have been carried out in simulated conditions [3]. Owing to methodological constraints, the extrapolation of the results obtained in these studies to real competition is difficult to assess [5]. There are a few studies which have investigated alpine skiing performance in competition, but this has mainly been carried out with biomechanical analysis of videos [6,7,8].

The use of Global Navigation Satellite Systems (GNSS) technology in particular is growing in alpine skiing [4,9,10,11], since its inception into the sport in 1997 [12]. This technology presents a big advantage owing to its precision in detecting substantial differences in skiers’ trajectories, causing minimal interference with the athletes and permitting a large capture volume [11,13]. Previous studies have had skiers use helmet mounted GNSS devices weighing almost 1kg to accurately measure the skier’s movements [10,14], but in other sports, studies have made the most of the advancement of technology by using lightweight, portable.

GNSS devices (approximately 8.0 × 4.0 ×1.5 cm) weighing less than 10 g which have in-built accelerometers. This has permitted comprehensive and real-time analysis of athletes’ performance during competition [15] and the assessment of training and match load on athletes [16]. Such devices not only help determine the kinematics of the skiers, but also give an idea about the impact loads on a skier during training competition. Although there have been studies that have looked into the performance using GNSS technology at the kinematic and kinetic level [4], a majority of research has focused on simulated conditions in male skiers [3]. Therefore, the objective of this study was to describe the kinematic patterns and impacts in male and female skiers during the course of an alpine skiing competition using a portable GNSS technology.

## 2. Materials and Methods

### 2.1. Study Design

A descriptive, cross-sectional research design was followed.

### 2.2. Participants

Fifteen athletes, nine male skiers (age 23.22 ± 2.19 years, weight 74.33 ± 3.46 kg and height 185.22 ± 6.33 cm) and six female skiers (age 21.53 ± 2.07 years, weight 63.6 ± 4.22 kg and height 167.83 ± 3.60 cm) volunteered to participate in this study. The skiers had experience at the international level having competed previously in the World Cup and European Cup. The data collection procedure was explained to the participants, and they provided written informed consent about their participation in the study. The study was conducted in accordance with the Declaration of Helsinki and approved by Ethics Committee (UE CIPI/19/151).

### 2.3. Disciplines

The athletes’ performance was recorded during the 2019 Spanish National championships in three disciplines: super-giant slalom, giant slalom and slalom. All the races were governed by the FIS rules through the international ski competition rules 2018. All competitions took part at the same time both environmental and meteorological conditions remained stable with sunny skies, subzero temperatures with very light wind and with hard frozen snow. 

All athletes belonged to the first group consisting of 15 athletes who drew their starting position on the previous day at the official race meeting. The course setter was chosen among the coaches of the national teams. 

The slope where the Spanish National Championship 2019 (Table 2) was held is a FIS homologated course for the development of international events for both men and women. The homologation was approved in 2015 following the International Competition Rules (ICR) standards of the FIS with approval number 11601/01/15. 

### 2.4. Procedure

**Figure 1:**
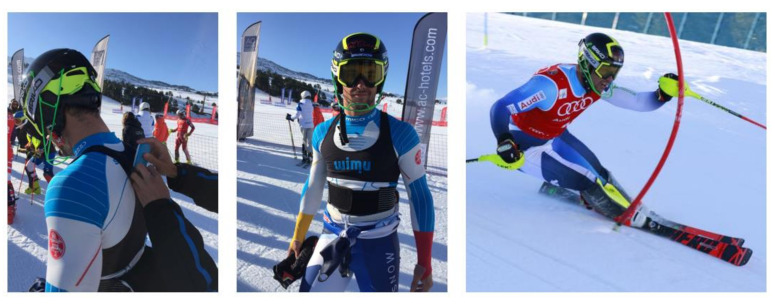
The placement of the Global Navigation Satellite Systems (GNSS) device on the vest on the skier.

Data acquisition in the different ski disciplines (super-giant slalom, giant slalom and slalom) was carried out using a wireless inertial measurement device (WIMUTM PRO, RealTrack Systems, Almería, Spain). Each skier was equipped with a customized vest with a pocket in the inter-scapular area where the GNSS device was inserted (Figure 1). The device is a validated [17], hybrid location system GNSS at 18 Hz with precision locator UWD (<10 cm) and 3D accelerometers 1000 Hz (400 g), with a weight of 60 g. 

The device was placed after the inspection of the track so that the subjects could do a warm-up with it and become adapted to the sensations of wearing it. All the participants confirmed that they did not cause any discomfort or perceived that they were wearing it. A researcher located at the start turned on the device 20 min before the start of the competition, with the skier staying still until the GNSS signal was located, while another investigator turned it off when the skier arrived at the finish line. The data obtained by the devices were synchronized and extracted for subsequent analysis using the manufacturer’s software SPRO™ (RealTrack Systems^®^, Almería, Spain).

### 2.5. Data Analysis

For each skier, the total distance covered was measured. This was divided into the different sectors based on the event (super-giant slalom and giant slalom had three sectors, while slalom had two). The distances covered in each sector was divided based on different speed thresholds: 35–60 km·h^−1^, ranges of 5 km·h^−1^ from 60 km·h^−1^ to 105 km·h^−1^ and >105 km·h^−1^ for super-giant slalom; 35–60 km·h^−1^, ranges of 5 km·h^−1^ from 60 km·h^−1^ to 85 km/h, and >85 km·h^−1^ for giant slalom; and 25.1–35 km·h^−1^, ranges of 5 km·h^−1^ from 60 km·h^−1^ to 70 km·h^−1^ for slalom. Additionally, the distances covered in these sectors during accelerations and decelerations were recorded, and these were classified in three categories: low accelerations or decelerations (between 0 and ±1.75 m·s^−2^), medium accelerations or decelerations (between ±1.76 and ±3 m·s^−2^) and high accelerations or decelerations (above ±3 m·s^−2^).

Impacts were measured by G-forces that the skiers experienced during their movements during the competition. This value represents the sum of the vector of G-forces the skier experienced in the vertical, antero-posterior, and medio-lateral axes. The impact value was registered when the G-forces of the movement exceeded 5 g [18,19,20]. 

The mean and standard deviation of the data were calculated. Shapiro Wilk’s test was used to test the normality of the data, and none of the variables satisfied the normal distribution criteria (*p* < 0.05 for all); non-parametric statistical techniques were used. The relationship between each variable and skier’s final ranking in each event was calculated using Spearman’s correlation coefficients (ρ), with the thresholds of ±0.1, ±0.3, ±0.5, ±0.7 and ±0.9 were considered to represent weak, moderate, strong, very strong and extremely strong positive/negative correlations, respectively. Non-parametric Mann–Whitney U-tests were used to compare the number of impacts between male and female skiers in the slalom and giant slalom disciplines. The effect sizes were determined using Cohen’s d, where the thresholds for small, moderate and large effects were 0.2, 0.5 and 0.8, respectively. The differences in the number of impacts per event in the case of female skiers were analyzed using non-parametric Mann–Whitney U-tests, and the effect sizes were determined using Cohen’s d. In the case of male skiers, Kruskal–Wallis one-way analysis of variance was used and in case significant differences were obtained pairwise comparisons were carried out with the Dwass–Steel–Critchlow–Fligner procedure. Effect sizes were calculated using Epsilon square (ϵ^2^) values, where the thresholds for weak, moderate and strong effects were 0.01, 0.04 and 0.16, respectively. Analyses were performed using Jamovi statistical package (version 1.2.17; www.jamovi.org, access date: 22/01/2021) with the threshold *p*-value for statistical significance set at *p* < 0.05.

## 3. Results

### 3.1. Super-Giant Slalom

**Figure 2:**
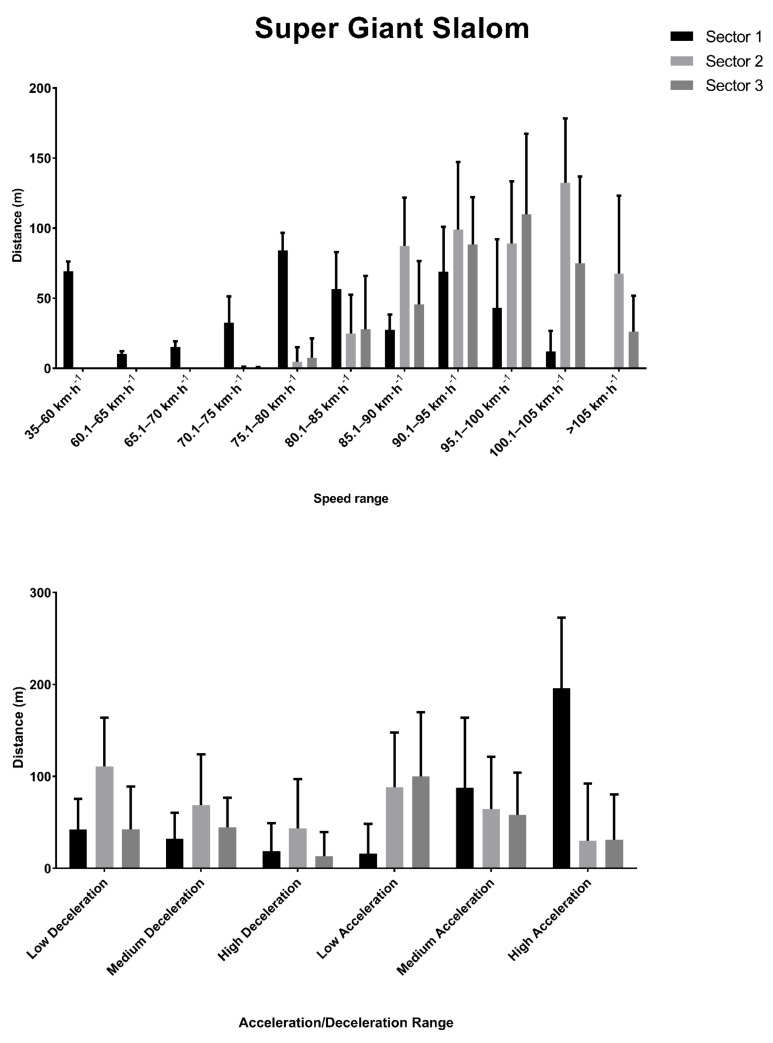
Distance covered in different speed and acceleration thresholds across the three sectors in the super-giant slalom event.

All nine male competitors completed the super-giant slalom event of the competition. The total distance covered by them was 1341.17 ± 40.88 m. On a sector-by-sector basis, the distance covered in Sector 1 was 444.40 ± 14.48 m, 510.79 ± 11.89 m in Sector 2 s and 385.97 ± 20.12 m in Sector 3. The distances covered in these sectors at the different speed and acceleration thresholds are shown in Figure 2.

The correlation analysis between the ranking and the other variables showed that a strong negative correlation coefficient was found between ranking and the total distance traveled (ρ = −0.883, *p* = 0.002) and the distance traveled over 105 km·h^−1^ (ρ = −0.767, *p* = 0.010), while the ranking had a strong positive correlation with the distance traveled between 75 and 80 km·h^−1^ (ρ = 0.685, *p* = 0.029).

### 3.2. Giant Slalom

**Figure 3:**
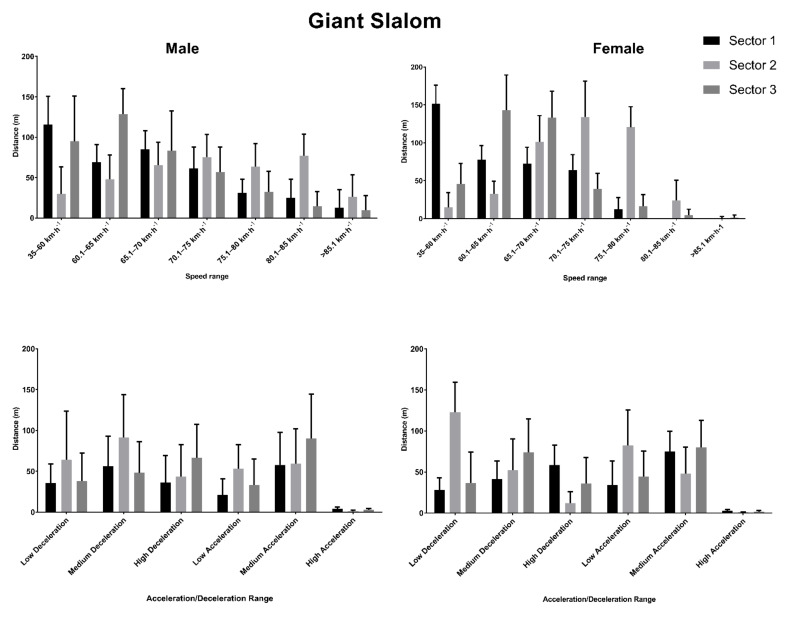
Distance covered in different speed and acceleration thresholds across the three sectors in the giant slalom event.

Seven male and six female skiers completed the two runs of the giant slalom event of the competition, while two male competitors completed a single run. The average total distance covered by the males was 1242.10 ± 41.73 m while for females was 1229.15 ± 27.23 m. On a sector-by-sector basis, the distance covered in Sector 1 was 422.15 ± 46.82 m for males and 399.88 ± 11.86 m for females, in Sector 2 it was 394.24 ± 95.10 m for males and 437.66 ± 58.59 m for females, and in Sector 3 it was 425.70 ± 35.01 m for males and 391.61 ± 52.01 m for females. The distances covered in these sectors at the different speed and acceleration thresholds are shown in Figure 3.

In male skiers, regarding the correlations of the skiers between the ranking and the variables, medium to strong negative correlations were found for distance travelled between 75 and 80 km·h^−1^ (ρ = −0.575, *p* = 0.020), distance travelled at 85–90 km·h^−1^ (ρ = −0.499, *p* = 0.049) and the total distance (ρ = −0.786, *p* < 0.001). On the other hand, the ranking in female skiers strongly correlated to the distance travelled at medium acceleration (ρ = 0.721, *p* = 0.008).

### 3.3. Slalom

**Figure 4:**
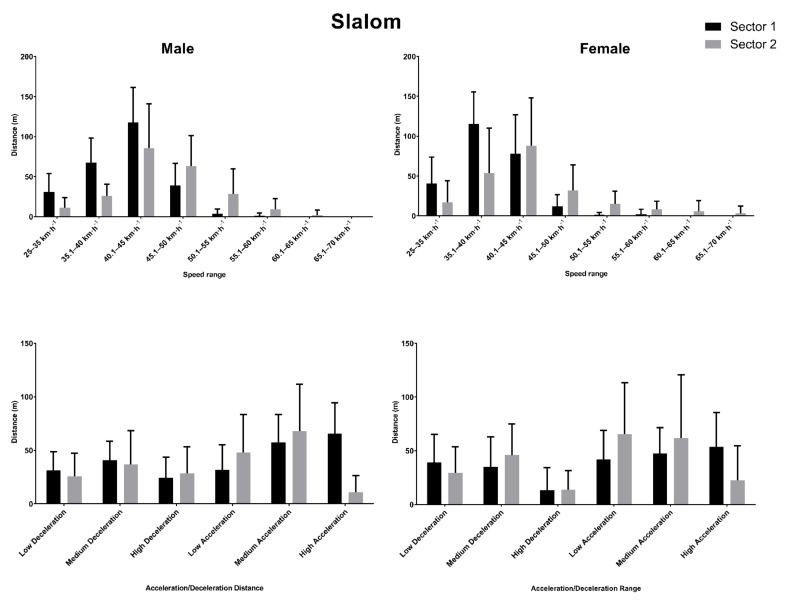
Distance covered in different speed and acceleration thresholds across the two sectors in the Slalom event.

Six male and three female skiers completed the two runs of the slalom event of the competition, while three male and three female competitors completed a single run. The total distance covered by the males was 532.74 ± 117.29 m, while for females it was 570.56 ± 133.49 m. On a sector-by-sector basis, the distance covered in Sector 1 was 275.66 ± 31.80 m for males and 275.32 ± 63.28 m for females, and in Sector 2 it was 257.07 ± 99.99 m for males and 297.34 ± 84.22 m for females. The distances covered in these sectors at the different speed and acceleration thresholds are shown in Figure 4.

The correlation analysis of the skiers’ ranking and the variables measured showed moderate to strong positive correlations in males for distances traveled between 25 and 35 km·h^−1^ (ρ = 0.589, *p* = 0.027) and 35–40 km·h^−1^ (ρ = 0.746, *p* = 0.002); and moderate to strong negative correlations between 50 and 55 km·h^−1^ (ρ = −0.562, *p* = 0.036), 55-60 km·h^−1^ (ρ = −0.701, *p* = 0.005), 60–65 (ρ = −0.537, *p* = 0.048) and for the total distance (ρ = −0.551, *p* = 0.041). No significant correlations were found for distances travelled at different velocity and acceleration thresholds in female skiers. 

### 3.4. Impacts

**Figure 5:**
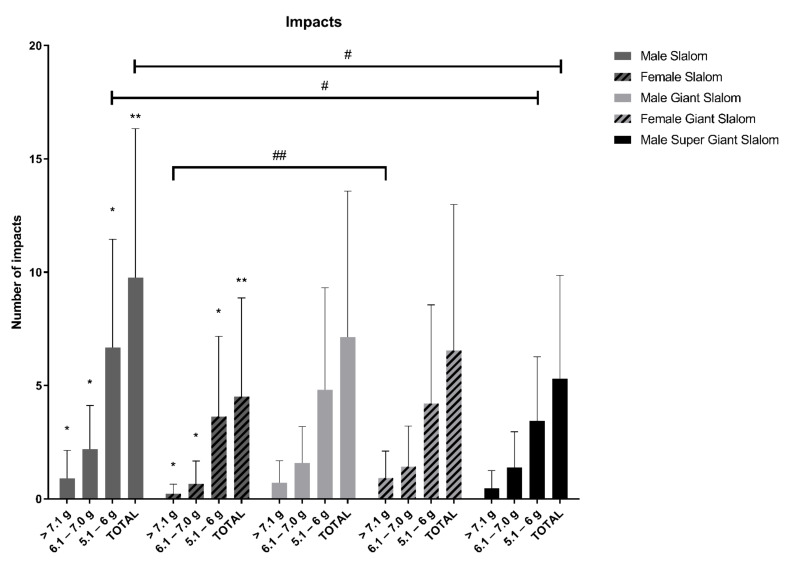
The number of impacts >5 g registered in slalom, giant slalom and super-giant slalom. Significant differences between male and female skiers in the same discipline are indicated by * at *p* < 0.05, and ** at *p* < 0.001. Significant differences between impacts in different disciplines for male and female skiers are indicated by # at *p* < 0.05, and ## at *p* < 0.001.

Comparing the number of impacts (Figure 5), differences were found between male and female skiers in slalom (total impacts: *p* < 0.001, 95% CI of difference = −8.10 to −2.32, d = 0.902; impacts > 7 g: *p* = 0.013, 95% CI of difference = −1.17 to −0.19, d = 0.686; impacts 6.1–7 g: *p* = 0.002, 95% CI of difference = −2.33 to −0.72, d = 0.934; impacts 5.1–6 g: *p* = 0.006, 95% CI of difference = −5.18 to −0.90, d = 0.699), but no differences were found in giant slalom (total impacts: *p* = 0.554; impacts > 7 g: *p* = 0.055; impacts 6.1–7 g: *p* = 0.664; impacts 5.1–6 g: *p* = 0.286). Keeping these differences in mind, the comparison between the number of impacts per discipline was separated based on sex. 

In male skiers, differences between disciplines were different for the total number of impacts (χ^2^ = 10.11, df = 2, *p* = 0.006, ϵ^2^ = 0.072) and impacts between 5.1 and 6g (χ^2^ = 9.64, df = 2, *p* = 0.008, ϵ^2^ = 0.068). Dwass–Steel–Critchlow–Fligner pairwise comparisons found greater impacts in slalom compared to super-giant slalom (total impacts: W = 4.43, *p* = 0.005; impacts 5.1–6 g: W = 4.31, *p* = 0.006). No significant differences were obtained for impacts in the range of 6.1–7 g (χ^2^ = 3.76, df = 2, *p* = 0.153) and <7 g (χ^2^ = 2.37, df = 2, *p* = 0.306).

In female skiers, differences were found in impacts above 7 g (*p* < 0.001, 95%CI of difference = 0.30 to 1.08, d = 0.853), while no differences were obtained for total impacts (*p* = 0.240), impacts in the range 5.1–6 g (*p* = 0.776) or impacts in the range of 6.1–7 g (*p* = 0.062).

## 4. Discussion

The importance of knowing the different performance parameters that affect elite alpine skiers in competition is essential to develop new practical training methodologies. Hence, including new technologies that allow the registration of these outcomes becomes necessary in sport. Given the increasing participation and the close results in competition, coaches need technology that they can use on a daily basis that is effective, accurate and non-invasive [9], and lightweight systems equipped with GNSS and accelerometers appear to offer the best technology for training and competition. This paper aimed to quantify the competitive race parameters in an elite alpine skiing competition in the disciplines of slalom, giant slalom and super-giant slalom. The results indicate that the distances covered by the skiers in different speed thresholds have a direct bearing on their final ranking, and these correlations vary based on the discipline of alpine skiing—this being the first study in which this outcome has been evaluated. The results also indicate that male and female skiers have different impacts in a more technical discipline such as a slalom but show no differences in the giant slalom discipline. The number of impacts also vary based on discipline in both male and female skiers. 

The results of this study show that the distance covered by skiers during competition affects their final rankings, similar to results presented earlier [4,21,22]. The trajectories of the skiers and their ability to maintain speed are very important in obtaining better results, a finding which has been corroborated by previous research [3,5]. Speed dominant events such as the super-giant slalom required athletes to travel greater distances at higher speeds in order to obtain a better ranking, as evidenced by the strong negative correlations at speeds above 105 km·h^−1^ and the ranking. In the case of giant slalom, the negative correlation obtained in this study between covering greater distances at higher speeds (75–80 and 85–90 km·h^−1^) and ranking shows that if a skier is able to stay at peak he can ensure a better maintenance of average speed, and therefore a better ranking position as a result of better performance. On the other hand, as has already been seen in other studies, to maintain these high speeds the skier must cover larger trajectories, i.e., cover larger distances at higher speeds [5]. Similarly, in the case of male skiers in the slalom discipline, covering greater distances at higher speeds corresponds to a shorter time to complete the course. 

However, in the case of female skiers, covering a greater distance at moderate accelerations correlates with a worse ranking, as it could indicate a previous deceleration and a lack of maintenance of high speeds at which accelerations are minimal or zero. In other words, it is possible that these skiers accelerate and brake often without being able to sustain peak speeds. This is an important finding given that the impacts that male and female skiers undergo are different in the slalom discipline. This has an important implication in the training of female skiers, which, as the results indicate, ought to differ from that of male skiers.

Skiing as a sport has high physical demands as skiers receive impacts higher than five times their bodyweight [23], a finding reiterated in this study. This implies that the strength of lower limb muscles is essential to improving performance in alpine skiing [24]. However, these impacts vary based on sex and discipline, and this is an important finding that coaches and trainers must consider given that athletes normally participate in these three disciplines. The number and magnitude of impacts are related to the speed of the skier: the higher the speed, the greater the number of impacts, and higher the magnitude of impacts. More turns should technically imply greater demands, as seen by the results in male skiers, where a greater number of impacts were observed for slalom compared to super-giant slalom [9]. In slalom, the difference in speed between men and women could be the reason for those differences. However, more research is needed to corroborate these findings, especially in conditions of competition and involving more female skiers.

The sample of skiers that participated in this study (*n* = 15) might appear to be low, but is similar or greater in number compared to other studies that have studied elite alpine skiers. This paper presents a novel technological advancement by using a trunk based GNSS device and accelerometer, which weighed 60 g, being much lower than other GNSS equipment used in other studies worn on the skier’s helmet [9,10]. This presents less intrusion for skiers and more benefits for coaches and trainers, permitting real-time analysis of skiing performance in training and competition. These initial findings can be corroborated by using a greater sample of athletes, both female and male, and by including other disciplines of alpine skiing.

## 5. Conclusions

Male and female alpine skiers showed different physical parameters and impacts even though they competed in the same courses in the disciplines of slalom, giant slalom and super-giant slalom. In male skiers, the distances traveled at different speed thresholds has a direct relation to the ranking of skiers, but this ideal threshold decreases as the technicality of the discipline increases. In the case of female skiers, although no relation was seen with the speed thresholds, greater distances covered at medium accelerations improve skiing performance. The number of impacts in alpine skiing vary based on sex and discipline. The use of lightweight, non-intrusive devices using GNSS technology and accelerometers in training and competitions could be essential to develop sex-specific and discipline-specific training programs in alpine skiing.

## Figures and Tables

**Table 1:** Characteristics of the different events of alpine skiing in international competition.

Event	Runs	Approx Track Length (m)	Vertical Drop (m)	Number of Direction Changes (% of Vertical Drop)
Super-Giant Slalom	1	1950	400–600	At least 7%
Giant Slalom	2	960	250–450	11–15%
Slalom	2	580	140–220	30–35%

**Table 2:** Characteristics of the alpine skiing events at the Spanish National Championship 2019.

Event	Number of Sectors	Vertical Drop (m)	Number of Direction Changes	Number of Gates	Average Separation between Gates (m)	
					Vertical	Horizontal
Super-Giant Slalom	3	450	24	26	52	12
Giant Slalom	3	351	47	49	26.5	10
Slalom	2	219	65	67	8	4

## Data Availability

Data available on request.
